# Complete Genome Sequence of the Citrobacter freundii Type Strain

**DOI:** 10.1128/MRA.00240-20

**Published:** 2020-05-07

**Authors:** Kenneth H. Wan, Soo Park, Becky M. Hess, Michael J. Neff, Benjamin W. Booth, Susan E. Celniker

**Affiliations:** a Biological Systems and Engineering Division, Lawrence Berkeley National Laboratory, Berkeley, California, USA; b Chemical and Biological Signatures, Pacific Northwest National Laboratory, Richland, Washington, USA; Loyola University Chicago

## Abstract

Citrobacter freundii is a species of facultative anaerobic Gram-negative bacteria of the family *Enterobacteriaceae*. The complete genome is composed of a single chromosomal circle of 4,957,773 bp with a G+C content of 52%.

## ANNOUNCEMENT

Citrobacter freundii is ubiquitous in soil and water and plays an important role in nitrogen fixation in the environment (1, 2). Although it is considered a commensal bacterium of the human intestinal microbiota, it is also an opportunistic pathogen causing infections in humans (3). Importantly, it contains genes that provide multidrug resistance to a broad spectrum of β-lactam antibiotics (4). With limited information regarding the diversity and physiology of C. freundii, it was important to sequence the genome of the Citrobacter freundii type strain obtained directly from the American Type Culture Collection (strain ATCC 8090), which was originally isolated from canal water (5).

Single colonies were isolated and grown in LB at 37°C. Genomic DNA isolated using Qiagen kits (catalog numbers 19060 and 10262) was sequenced on three platforms, namely, Pacific Biosciences (PacBio), Nanopore, and Illumina platforms. Genomic DNA was sent to GENEWIZ for PacBio library construction and sequencing. Libraries (∼10 kb) were constructed using SMRTbell template preparation v1.0, Sequel binding v2.0, and MagBead v2 kits and sequenced on a PacBio Sequel instrument using software v5.0.0.6235 with 10-hour movie times. Default parameters were used for all software tools.

PacBio sequencing yielded a total of 635,329 reads with an *N*_50_ value of 5,267 bp, totaling 2.6 Gbp. A *de novo* assembly was constructed using Canu v1.7 (6). Sequence quality was assessed using Minimap2 (7). The percentage of reads mapping to the genome is 96.82%, and the estimated coverage is 508×.

Nanopore sequencing was performed using the rapid barcoding kit (SQK-RBK004), run with a MinION flow cell (FLO-MIN106D) using MinKNOW v1.13.1, and reads were base called using Guppy v2.0.10. Nanopore sequencing yielded a total of 462,441 reads with an *N*_50_ value of 8,149 bp, totaling 1.9 Gbp.

Illumina libraries were prepared with the NEBNext Ultra DNA and multiplex oligomer kits (catalog numbers E7370 and E7600) and run on a MiSeq system with v3 chemistry (catalog number MS-102-3003) with paired-end sequencing (2 × 300 nucleotides). Illumina sequencing yielded a total of 4,421,404 reads passing filter, totaling 1.3 Gbp. The Nanopore and Illumina reads were jointly assembled using Unicycler v0.4.8-beta (8) with 94.16% and 99.95% reads mapping, respectively, and estimated coverages of 358× and 269×, respectively. Since the assemblies are virtually identical (99.99% average nucleotide identity [ANI]) (9), we analyzed and annotated the Unicycler assembly using RAST v2.0 (10) and NCBI Prokaryotic Genome Annotation Pipeline v4.11 (11). The annotation predicts 4,734 genes in total, with 4,550 protein-coding genes and 119 RNA genes, including 8 rRNA operons, 1 singleton small-subunit rRNA (5S rRNA), 11 noncoding RNAs, and 83 tRNA genes, 1 of which is a tRNA-SeC(p)-TCA. Over 170 genes are contained within four candidate prophages (27.7 kb, 32.2 kb, 24.4 kb, and 38.5 kb), representing 2.5% of the genome. As expected from the canal water isolation of this species, KEGG analysis (12) shows that it contains 65 genes that encode and regulate flagellar assembly and function.

Our sequence is similar (ANI, 99.98% [9]) to that of C. freundii strain NCTC9750 (GenBank accession number LR134118) but is smaller by 2,964 bp, differing in the structure of a prophage and insertion of a transposase (InsH) gene. Our sequence also differs in the number of candidate nonessential gene mutations (65 versus 600 in strain NCTC9750).

The identification of tRNA-SeC(p)-TCA led us to search the genome for selenoprotein biosynthesis and metabolism genes. We identified a number of these genes, including *selA*, *selB*, *selD*, *ydf2*, *yedE*, *yedF*, and *ygeY*. At least three proteins likely contain selenocysteine, including those encoded by homologs of the gene for formate dehydrogenase (*fdnG*), a known SeC-containing protein (13). Further characterization of selenoproteins will provide new insights into the roles these proteins play in antioxidant and other activities.

### Data availability.

The chromosome sequence of C. freundii ATCC 8090 is deposited in GenBank under accession number CP049015. The SRA numbers are SRR11179868 and SRR11179867 for the Illumina reads, SRR11179866 and SRR11179865 for the Nanopore reads, and SRR11179864 for the PacBio reads. C. freundii *strain* NCTC9750 was analyzed for comparison.
